# A cross-sectional study on the effect of dietary zinc intake on the relationship between serum vitamin D_3_ and HOMA-IR

**DOI:** 10.3389/fnut.2022.945811

**Published:** 2022-10-24

**Authors:** Biao Hu, Zheng-yang Lin, Yuan Cai, Yue-xin Sun, Shu-qi Yang, Jiang-long Guo, Shi Zhang, Dong-lin Sun

**Affiliations:** ^1^Department of Clinical Medicine, The Second Clinical School of Guangzhou Medical University, Guangzhou, China; ^2^Department of Preventive Medicine, School of Public Health, Guangzhou Medical University, Guangzhou, China; ^3^Department of Medical Imaging, The Second Clinical School of Guangzhou Medical University, Guangzhou, China; ^4^Guangzhou Medical University, Guangzhou, China

**Keywords:** vitamin D3, zinc, insulin resistance, a cross-sectional study, interaction

## Abstract

**Introduction:**

Serum vitamin D_3_ concentration is associated with the risk of insulin resistance. Zinc has also been reported to be associated with a lower risk of insulin resistance. In addition, zinc is an essential cofactor in the activation of vitamin D_3_. However, the effect of dietary zinc intake on the relationship between vitamin D_3_ and insulin resistance risk has not been fully studied. Therefore, we designed this cross-sectional study to assess the impact of changes in zinc intake on the relationship between vitamin D_3_ and insulin resistance risk.

**Study design and methods:**

This study analyzed data from the national Health and Nutrition Examination Survey (NHANES) from 2005 to 2018, involving 9,545 participants. Participants were stratified by zinc intake category (low zinc intake <9.58 mg/ day; High zinc intake: ≥9.58 mg/ day).

**Results:**

In this cross-sectional study, serum vitamin D_3_ levels were independently associated with the risk of insulin resistance in both the low and high Zinc intakes (β: −0.26, 95%Cl: −0.56~0.04 vs. β: −0.56, 95%Cl: −1.01~-0.11). In addition, this association was influenced by different dietary zinc intakes (interaction *P* < 0.05).

**Conclusions:**

Our results suggest that zinc intake may influence the association between serum vitamin D_3_ and the risk of insulin resistance. Further randomized controlled trials are needed to provide more evidence of this finding.

## Introduction

Insulin resistance is a part of abnormal cardiovascular metabolism, often referred to as “insulin resistance syndrome” or “metabolic syndrome,” which may also accelerate the development of atherosclerosis, hypertension or polycystic ovary syndrome (1). Especially, Insulin resistance has been confirmed to be closely related to the onset of type 2 diabetes (T2D) (2). The increase in the prevalence of T2D has become a serious public health problem, resulting in an increase in related morbidity and mortality in addition to a huge economic burden (3). Therefore, it is necessary to identify the nutrients associated with insulin resistance to prevent insulin resistance.

Vitamin D deficiency is an epidemic (4) and has been linked to asthma, diabetes, cancer and neuropsychiatric disorders (5). In recent years, there have been numerous studies on the relationship between vitamin D and insulin resistance (6–9). However, the relationship between vitamin D_3_ and insulin resistance remains controversial. The study of Mahendra Bhauraoji Gandhe et al. showed a significant negative correlation between vitamin D status and insulin level, suggesting that vitamin D supplementation may increase insulin sensitivity (10). However, the study of Zixin Xu et al. showed that such correlation varies between individuals and races (11). In addition, oral glucose tolerance tests conducted by Dilek Erdonmez et al. in some high school students showed no correlation between insulin measurements and vitamin D deficiency (12). These differences in findings may be due to potential confounding factors that have not been fully considered, such as dietary zinc intake.

Zinc has been reported to be associated with insulin resistance risk. Higher serum zinc concentrations are associated with increased insulin sensitivity (13). Zinc has specific functions in the biochemistry of insulin and glucagon in pancreatic β- and α- cells (14). In addition, the expression of the gene SLC30A10 encoding the zinc transporter ZnT10 was regulated by vitamin D_3_ (15). It is concluded that zinc may be associated with the activation of vitamin D. However, the current study has not fully explored the effect of dietary zinc intake on the relationship between vitamin D_3_ and insulin resistance risk. Therefore, in this cross-sectional study, we hypothesized that zinc and vitamin D_3_ interact with insulin resistance. We aimed to investigate the effect of zinc intake on the association between vitamin D_3_ and insulin resistance.

## Methods

### Data sources and study population

This is a cross-sectional study. We used data from the National Health and Nutrition Examination Survey (NHANES) continuously from 2005 to 2018. Participants included in the analysis were aged 20 years or older and had completed interviews and examinations in the mobile examination center (MEC). Participants with unknown serum vitamin D, insulin resistance, and covariates were excluded. NHANES (16) is a national health-related survey designed to assess the health and nutritional status of non-hospitalized US citizens. Survey participants selected representative samples of multi-stage stratified probability indicators (17). Extensive household interviews were conducted to gather demographic and health history information. A physical examination was performed and blood samples were collected in the MEC. The serum samples were analyzed in the United States by the Laboratory Science Division of the National Center for Environmental Health and the Centers for Disease Control and Prevention.

The study was approved by the Ethics Review Board of the National Center for Health Statistics Research. Before starting the study, the protocol was approved by the ethics board of the national review board CPP Sud-Méditerranée IV. Our research is based on public data from the NHANES, all details are from the official website (https://www.cdc.gov/nchs/nhanes/index.htm).

### Zinc intake

Data on zinc dietary intake during the previous 24 h were collected through MEC's dietary review interview. Daily zinc intakes were divided into high and low intakes based on a median (9.58 mg/d). In large-scale surveys, 24-h recall (18) is the most commonly used dietary intake survey method. The decision to continue using the method at NHANES over the years was based on a consensus reached by the expert group at regular seminars to evaluate NHANES 'data collection methods (19).

### Definition of insulin resistance

HOMA-IR is used to evaluate individual insulin resistance levels. The index was calculated as follows: fasting glucose level (FPG, mmol/L) × fasting insulin level (FINS, μU/mL) /22.5. Where, coefficient 22.5 is the correction factor, which refers to the blood glucose level of 4.5 mmol/L corresponding to 5 μU/mL plasma insulin in normal individuals. HOMA-IR reflects the interaction between glucose and insulin in different organs. The HOMA-IR index of normal individuals is 1. With the increase of insulin resistance level, the HOMA-IR index will be higher than 1.

### Covariates

This article takes into consideration the age, sex, race/ethnicity, marital status, PIR, BMI, high-density lipoprotein cholesterol (HDL-c), education level, smoking status, physical condition, activity, alcohol, level of serum vitamin D_3_, magnesium dietary intake, dietary zinc intake, dietary calcium intake, serum vitamin A level, total cholesterol, as A potential confounding factors. Race and ethnicity are divided into Mexican-Americans, non-Hispanic blacks, non-Hispanic whites, other Hispanics, and other races, including multiracial. Marital status is divided into married and unmarried, and married includes cohabitation, separation, divorce and widowhood. Smoking status is classified as current smokers, former smokers and never smokers. Participants who reported not smoking 100 cigarettes in their lifetime were considered never-smokers. Participants who smoked more than 100 cigarettes in their lifetime but did not currently smoke were considered former smokers. Reports have smoked more than 100 cigarettes in their lifetime and are now considered current smokers sometimes even daily. We define household income using the poverty income ratio (PIR), which is calculated from a specific threshold for household size. BMI is an internationally used measure of obesity and health, calculated from weight and height. Measure your weight in pounds on a digital scale and convert it to kilograms. Height is measured with an electronic tacheometer, accurate to millimeters. Education level includes below high school, high school graduation and university degree or above. Physical is classified as no or unknown, moderate or vigorous, according to whether moderate exercise caused a slight increase in respiration or heart rate, and vigorous exercise, fitness or recreational activities caused a significant increase in respiration or heart rate during the week. Work activity was classified according to three levels of activity intensity, non-work activity, moderate work activity and vigorous work activity. The drinking status of the drinkers and non-drinkers. Categorize drinkers as having more than 12 alcoholic drinks per year. Dietary recall interviews were conducted prior to MEC interviews to collect dietary information for the previous 24 h, including total dietary energy, vitamin D_3_, magnesium, zinc, and calcium. Vitamin D_3_ is the concentration in the serum.

### Statistical analysis

All analyses were performed using statistical software package R (http://www.R-project.org, R Foundation). The relationship between serum vitamin D_3_ concentration and insulin resistance was compared between low and high zinc intakes. We used the sample weight provided by NHANES. A hierarchical weighted multiple logistic regression model was used to subgroup zinc intake. Calculate the beta value and 95% confidence interval. The likelihood ratio test was used to examine the interactions between subgroups. A linear trend test was performed by entering the median value of each serum Vitamin D_3_ as a continuous variable in the model. *P*-values < 0 are considered statistically significant. The continuous variables are analyzed by *t*-test (Normal distribution) and Kruskal–Wallis (skewness distribution) tests.

## Result

### Baseline characteristics of the study population

**Figure 1:**
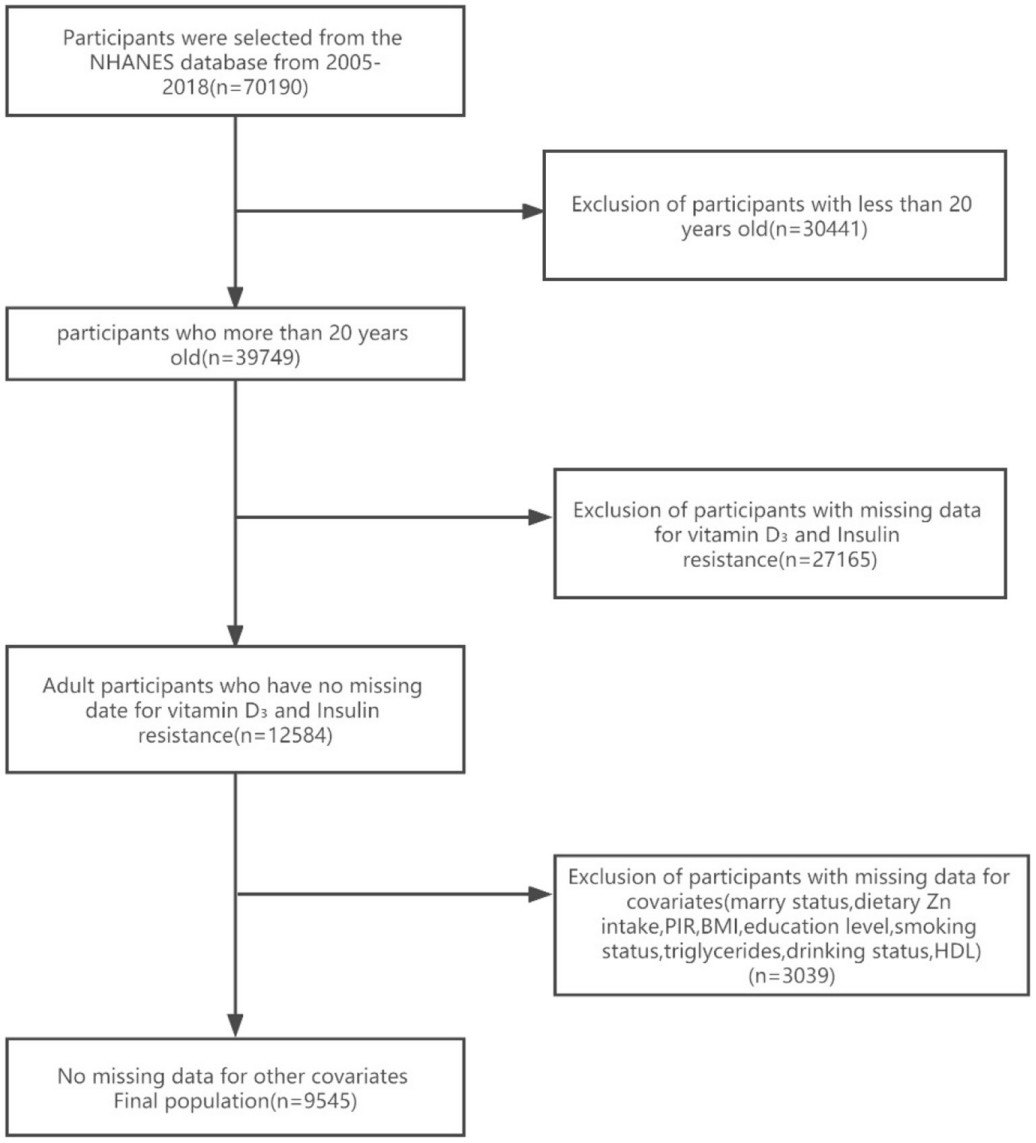
The flow chart of the study.

Seven NHANES cycles were used in this study, namely 2005–2006, 2007–2008, 2009–2010, 2011–2012, 2013–2014, 2015–2016, and 2017–2018. As shown in the flow chart (Figure 1), 70,190 potential participants were identified; among them, 39,749 adults (≥20 years old) were included, excluding minor participants younger than 20 years old (*n* = 30,441); Participants with absent serum 25-hydroxyvitamin D concentration data and absent insulin resistance were excluded (*n* = 27,165). After excluding participants with missing covariant data, the remaining 9,545 participants were included in our analysis. A flowchart for exclusion criteria is shown in Figure 1. Table 1 shows the baseline characteristics of a study population divided into two groups based on dietary zinc intake. Individuals with high zinc intakes (≥9.58 mg/day) were more likely to be male, Caucasian, younger, stronger, more physically active, more highly educated, and mostly with higher incomes than those with low zinc intakes (< 9.58 mg/day). Individuals with high zinc intake drank less alcohol and had lower HDL levels. In terms of dietary factors, dietary calcium, magnesium, vitamin D_3_ and vitamin A intakes were higher among participants with higher zinc intakes. There were no significant differences in marital status, BMI, smoking status and HDL levels between the high and low zinc intake groups.

**Table 1:** Baseline characteristics of participants.

	**Dietary zinc intake (mg/d)**			
**Variables**	**Total (*n* = 9,545)**	**Zinc ≤ 9.58 (mg/d) (*n* = 4,769)**	**Zinc > 9.58 (mg/d) (*n* = 4,776)**	***p***
**Age, median (IQR)**	50.0 (34.0, 64.0)	52.0 (36.0, 66.0)	47.0 (34.0, 62.0)	< 0.001
**Gender**, ***n*** **(%)**				< 0.001
Female	4,852 (50.8)	2,957 (62)	1,895 (39.7)	
Male	4,693 (49.2)	1,812 (38)	2,881 (60.3)	
**Race**, ***n*** **(%)**				< 0.001
Mexican American	1,415 (14.8)	638 (13.4)	777 (16.3)	
Non-Hispanic black	1,821 (19.1)	1,058 (22.2)	763 (16)	
Non-Hispanic white	4,405 (46.1)	2,050 (43)	2,355 (49.3)	
Other Hispanic	993 (10.4)	548 (11.5)	445 (9.3)	
Other race	911 (9.5)	475 (10)	436 (9.1)	
**Marital status**, ***n*** **(%)**				0.755
No	1,724 (18.1)	855 (17.9)	869 (18.2)	
Yes	7,821 (81.9)	3,914 (82.1)	3,907 (81.8)	
**PIR, Mean ±SD**	2.5 ± 1.6	2.4 ± 1.6	2.6 ± 1.6	< 0.001
**BMI**, ***n*** **(%)**				0.158
< 25	2,791 (29.2)	1,412 (29.6)	1,379 (28.9)	
25–29.9	3,221 (33.7)	1,565 (32.8)	1,656 (34.7)	
>30	3,533 (37.0)	1,792 (37.6)	1,741 (36.5)	
**HDL, Mean ±SD**	54.1 ± 16.0	55.4 ± 16.6	52.8 ± 15.2	< 0.001
**Education level**, ***n*** **(%)**				< 0.001
Less than high school	2,267 (23.8)	1,249 (26.2)	1,018 (21.3)	
High school graduation	2,161 (22.6)	1,103 (23.1)	1,058 (22.2)	
College or above	5,117 (53.6)	2,417 (50.7)	2,700 (56.5)	
**Smoking status**, ***n*** **(%)**				0.004
Never	5,244 (54.9)	2,640 (55.4)	2,604 (54.5)	
Former	2,376 (24.9)	1,123 (23.5)	1,253 (26.2)	
Now	1,925 (20.2)	1,006 (21.1)	919 (19.2)	
**Physical activity**, ***n*** **(%)**				< 0.001
No/Unknown	4,923 (51.6)	2,616 (54.9)	2,307 (48.3)	
Moderate	2,573 (27.0)	1,311 (27.5)	1,262 (26.4)	
Vigorous	2,049 (21.5)	842 (17.7)	1,207 (25.3)	
**Work activity**, ***n*** **(%)**				< 0.001
Non-work activity	5,556 (58.2)	2,935 (61.5)	2,621 (54.9)	
Moderate work activity	2,154 (22.6)	1,058 (22.2)	1,096 (22.9)	
Vigorous work activity	1,835 (19.2)	776 (16.3)	1,059 (22.2)	
**Alcohol**, ***n*** **(%)**				< 0.001
Yes	2,619 (27.4)	1,504 (31.5)	1,115 (23.3)	
No	6,926 (72.6)	3,265 (68.5)	3,661 (76.7)	
**Serum Vitamin D**_**3**_ **, Mean ±SD**	60.8 ± 27.0	59.4 ± 28.4	62.2 ± 25.4	< 0.001
**Magnesium intake, Mean ±SD**	292.5 ± 147.3	218.0 ± 91.8	366.8 ± 154.6	< 0.001
**Zinc intake, Mean ±SD**	11.1 ± 7.0	6.4 ± 2.0	15.8 ± 7.0	< 0.001
**Calcium intake, Median (IQR)**	803.0 (519.0, 1163.0)	600.0 (398.0, 845.0)	1066.0 (750.0, 1469.0)	< 0.001
**Vitamin A intake, Median (IQR)**	459.0 (256.0, 764.0)	333.0 (176.0, 556.0)	621.5 (380.0, 939.2)	< 0.001
**Total chol, Mean ±SD**	191.8 ± 40.7	192.4 ± 41.3	191.2 ± 40.2	0.136

Data presented are ORs and 95% Cls. BMI, Body Mass Index; PIR, the ratio of family income to poverty; Total chol, total cholesterol.

### Zinc intake affects the association between vitamin D_3_ and insulin resistance

After adjusted for age, gender, race/ethnicity, BMI, education level, physical activity, smoking status, alcohol, PIR, marital status, HDL, total cholesterol, work activity, dietary magnesium intake, dietary calcium intake, dietary zinc intake, serum vitamin D_3_ levels, dietary vitamin A intake, and magnesium had an interaction with the association between vitamin D_3_ and insulin resistance (Table 2). Vitamin D_3_ was used as a categorical variable. The two groups were divided into two groups: low level group (≤58.3 nmol/L) and high level group (>58.3 nmol/L). In the high zinc intake group, the mean β value of insulin resistance of participants with serum vitamin D_3_ >58.3 nmol/L was −0.56 (95%CIS: −1.01–0.11, *P* = 0.014), suggesting a correlation between dietary zinc intake and vitamin D_3_ and insulin resistance (likelihood ratio test of interaction *P* < 0.05, *P* < 0.05). However, there was no significant difference in the low zinc intake group (*P* = 0.092). In addition, serum vitamin D_3_ levels were further divided into three groups: low level group (≤47.3 nmol/L), moderate level (47.3–69.8 nmol /L) and high level (≤69.8 nmol/L). Among participants with high zinc intake, there was an independent association between serum vitamin D_3_ levels and HOMA-IR index, and whether this association was affected by different zinc intake. Insulin resistance decreased significantly with increased vitamin D_3_ concentration in the high-zinc group (β = −1.04, 95%CI: −1.61 ~ −0.46, *P* < 0.001) but not in the low-zinc group (β = −0.27, 95%CI: −0.64 ~ 0.1, *P* = 0.154).

**Table 2:** Interactive effect of vitamin D_3_ and dietary zinc intake on insulin resistance (all participants).

**Variable**	**Dietary intake zinc** ≤ **9.58(mg/d)** **(*****n*** = **4,769)**		**Dietary intake zinc** > **9.58 (mg/d)** **(*****n*** = **4,776)**		***P*** **for interaction**
**Vitamin D_3_ (nmol/l)**	**β (95CI%)**	***P*** **-value**	**β (95CI%)**	***P*** **-value**	
**Subgroups-1**					0.034
≤58.3	0 (reference)		0 (reference)		
>58.3	−0.26 (−0.56~0.04)	0.092	−0.56 (−1.01~-0.11)	0.014	
**Subgroups-2**					0.001
≤47.3	0 (reference)		0 (reference)		
47.3–69.8	−0.21 (−0.56~0.13)	0.225	−1.02 (−1.55~-0.49)	< 0.001	
>69.8	−0.27 (−0.64~0.1)	0.154	−1.04 (−1.61~-0.46)	< 0.001	
**Trend test**	−0.14 (−0.32~0.05)	0.147	−0.5 (−0.79~-0.21)	0.001	

Adjusted for age, gender, race/ethnicity, BMI, education level, physical activity, smoking status, alcohol, PIR, marital status, HDL, total cholesterol, work activity, dietary magnesium intake, dietary calcium intake, dietary zinc intake, serum vitamin D_3_ levels, dietary vitamin A intake.

## Discussion

In a sample of adults over 20 years of age from the National Health and Nutrition Examination Survey (NHANES), our results suggest that serum vitamin D_3_ levels were significantly higher in non-insulin-resistant participants with high zinc intake than in insulin-resistant participants. Serum vitamin D_3_ concentration was inversely associated with insulin resistance risk in the high zinc intake group, but not in the low zinc intake group. We suspect that the correlation between serum vitamin D_3_ concentration and the risk of insulin resistance is only apparent when zinc concentration reaches a certain threshold. In addition, a relationship between dietary zinc intake and vitamin D in the treatment of diabetes was also found (20), suggesting that vitamin D adequacy and high zinc intake are greater than the sum of individual effects.

The main function of vitamin D_3_ is to regulate bone metabolism and calcium phosphate homeostasis. In addition, vitamin D_3_ may play an important role in maintaining pancreatic cell function, the study reports. It works by activating vitamin D receptors (VDR) and regulating insulin secretion through calcium channels located in pancreatic cells (9). Vitamin D_3_ plays a role in the prevention of insulin resistance by improving insulin secretion, glucose metabolism, glucose tolerance, insulin sensitivity and inhibiting systemic inflammation. Interaction of 1,25 (OH)2 vitamin D with nVDR leads to transcription of insulin, cell structure, and growth genes. Two prospective cohort studies support our conclusions, showing that higher vitamin D concentrations are inversely associated with the risk of insulin resistance. Another study showed that vitamin D can indirectly stimulate insulin secretion and reduce the risk of insulin resistance by normalizing extracellular calcium by changing the calcium flow of cell membranes (21). A study by Nagashima et al. showed that 1, 25-dihydroxyl metabolites metabolized by liver and kidney hydroxylase could prevent quitiapine-induced insulin resistance *in vitro* through the PI3K signaling pathway (8). In a large Canadian cohort of non-diabetic adults, vitamin D status was found to be inversely associated with insulin responsiveness. Insulin response was associated with 25(OH) vitamin D levels in patients with baseline 25(OH) vitamin D levels ranging from 40 to 90 nmol/L, after adjusting for BMI, waist circumference, body weight, age, and sex (22). In addition, plasma vitamin D levels were negatively associated with classic parameters of obesity such as body mass index, fat mass and waist circumference. Notably, serum 25(OH) vitamin D levels were significantly lower in obese people than in lean people. Overweight and obese people were 25 percent and 35 percent more likely to have vitamin D deficiency than lean people, respectively (6).

However, Burnett et al. showed no association between total vitamin D intake and type 2 diabetes after adjustment for multiple potential confounders (23). A study examining the association between vitamin D and diabetes in the Thai population showed that the association between vitamin D and HbA1c was observed only in certain age groups (35–74 years), and that vitamin D deficiency had a significant effect only on older subjects living in an urban environment (24). It can be speculated that the relationship between vitamin D and insulin resistance is greatly affected by environmental factors. Similarly, Sadyia et al. reported no significant change in HbA1c percentage in the UAE population after 6 months of vitamin D supplementation (25). This may be due to the large differences between our sample and those in the clinical trials or due to the fact that the samples in the clinical trials did not control the levels of other factors that may affect vitamin D_3_ absorption, thus influencing the effect of vitamin D_3_ on insulin resistance.

Zinc is an essential trace element and micronutrient in human body and plays an important role in various physiological processes. Human needs for zinc are second only to those for iron. Its deficiency was significantly associated with induced oxidative stress, inflammatory events, and vascular dysfunction. Epidemiological studies have shown that low serum zinc levels are negatively correlated with a variety of diseases, such as diabetes, coronary artery disease and Parkinson's disease (26–32). Since 1934, when zinc was shown to be a component of insulin crystals, a link between zinc and diabetes has been proposed. Zinc plays a key role in insulin secretion and signaling. One study suggests that changes in biochemical parameters of zinc observed in obese individuals contribute to the presentation of related diseases, such as insulin resistance (8). In addition, several studies have established that zinc plays a fundamental role in insulin synthesis, storage, and action by stimulating its receptor, which protects liver and pancreatic cells from free radicals. Furthermore, as a nutrient that plays an important role in insulin sensitivity, zinc plays an indispensable role in maintaining the stability of insulin Homer (33). According to Jansen et al., disturbance of zinc homeostasis seems to be associated not only with diabetes, but also with several other diseases, such as cirrhosis, tumor, intestinal disease, and impaired immune system function (34).

A recent animal trial exploring the relationship between zinc supplementation and hyperglycemia and associated metabolic abnormalities revealed that following zinc supplementation, diabetic rats had a significant increase in plasma albumin, decrease in plasma urea and creatinine levels, and significant changes in insulin sensitivity indices HOMA-IR, HOMA-B, and QUICKI. Thus, this experiment provides the first evidence indicating that zinc supplementation can partially ameliorate the severity of diabetic hyperglycemia and the associated metabolic abnormalities, hypoinsulinemia, insulin resistance, and morphological changes of the pancreas, thereby inferring that zinc supplementation may offer significant potential for clinical applications in managing diabetic hyperglycemia and associated metabolic complications (35) which is consistent with our findings. Another study also revealed that zinc oxide nanoparticles act as effective antidiabetic agents (36).

Vitamin D_3_ can directly affect cellular zinc homeostasis by inducing zinc transporters. In a study of cells treated with vitamin D, there was a 15 fold increase in the SLC30A10 gene, which is responsible for the translation of the zinc transporter znt10 protein. Vitamin D_3_ can significantly alter the expression of various transporter genes involved in multiple physiological processes, including drug metabolism and transport, in Caco-2 cells. The SLC30A10 gene and its encoded protein znt10 were increased in Caco-2 cells treated with vitamin D_3_, which may indicate a molecular correlation between vitamin D_3_ levels and zinc regulation (15).

Given the prevalence and widespread supplementation of vitamin D deficiency, it would be interesting to understand the physiological and pharmacological implications of vitamin D_3_ induction of SLC30A10/ZnT10. In theory, the end result would be a decrease in zinc levels in the cytoplasm, with an increase in zinc levels in the stored organelles and extracellular fluid. It is tempting to speculate that vitamin D_3_ stimulates the release of these metal ions into the circulation, reaching organs in need, and helps vitamin D_3_ perform some physiological functions. For example, vitamin D_3_, zinc and manganese have bone protective effects and can stimulate bone formation (37). Zinc is considered as a supplement for the treatment and prevention of osteoporosis (38). It is not known whether there is a synergistic effect between vitamin D_3_, zinc and manganese, but some pathological symptoms may indicate an important link between these nutrients. Vitamin D and zinc levels are particularly reduced in inflammatory bowel disease (IBD), celiac disease, and food-protein induced gastrointestinal allergy (FPGIA) (39–41). Although malabsorption is a hallmark of these intestinal diseases, the regulatory role of these nutrients, in which an imbalance in one nutrient affects the circulating concentration of another, cannot be ruled out. Maternal intake of vitamin D and zinc, as well as vitamin E deficiency, is also associated with childhood asthma (42), and monitoring and supplementation of zinc and vitamin D is considered as a potential way to control wheezing in children (43). Our current data confirm previous reports and reveal that vitamin D_3_ plays some role in zinc homeostasis. Our current data confirm previous reports and reveal that vitamin D_3_ plays some role in zinc homeostasis.

The study has some limitations. First, as a cross-sectional observational study, the associations found in this study may not lead to direct causation and may be confused by some other unmeasured variables. However, a number of potential confounding factors, including some dietary factors, were adjusted for in the logistic regression model. Second, although we used a large sample, the study was limited to US residents. Therefore, when extrapolating to other groups, it is necessary to take this aspect into due consideration. Third, recall and self-report bias may occur because dietary data were obtained from self-reported 24-h dietary recall. The same participant may result in inaccurate results if the sample is re-sampled at different times.

## Conclusion

To sum up, although there is evidence that vitamin D_3_, zinc, and there was a link between insulin resistance, but the existing data are not sufficient to prove that a lack of vitamin D_3_ in reducing insulin resistance and related role in the pathogenesis of the metabolic syndrome and the general reasons, may not be enough to prove that vitamin D_3_ supplements to treat insulin resistance and metabolic syndrome. We believe that long-term, well-designed interventional clinical trials should be initiated to better understand the therapeutic potential of vitamin D_3_ supplementation in insulin-resistant subjects with vitamin D_3_ deficiency, focusing on dosage, treatment duration, side effects, and short- and long-term outcomes.

## Data availability statement

Publicly available datasets were analyzed in this study. This data can be found here: https://www.cdc.gov/nchs/nhanes/index.htm.

## Author contributions

Thanks to BH, Z-yL, and YC for their contribution to data processing and topic selection, at the same time, thanks to Y-xS and S-qY for their contribution in writing. I am also grateful to J-lG for his contribution to consulting relevant literature. All authors contributed to the article and approved the submitted version.

## Conflict of interest

The authors declare that the research was conducted in the absence of any commercial or financial relationships that could be construed as a potential conflict of interest.

## Publisher's note

All claims expressed in this article are solely those of the authors and do not necessarily represent those of their affiliated organizations, or those of the publisher, the editors and the reviewers. Any product that may be evaluated in this article, or claim that may be made by its manufacturer, is not guaranteed or endorsed by the publisher.

## References

[1] Lebovitz HE. Insulin resistance: definition and consequences. Exp Clin Endocrinol Diabetes. (2001) 109(Suppl. 2):S135–48. 10.1055/s-2001-18576 11460565

[2] Petersen MC Shulman GI. Mechanisms of insulin action and insulin resistance. Physiol Rev. (2018) 98:2133–223. 10.1152/physrev.00063.2017 30067154 PMC6170977

[3] Wu Y Ding Y Tanaka Y Zhang W. Risk factors contributing to type 2 diabetes and recent advances in the treatment and prevention. Int J Med Sci. (2014) 11:1185–200. 10.7150/ijms.10001 25249787 PMC4166864

[4] Dobnig H. A review of the health consequences of the vitamin D deficiency pandemic. J Neurol Sci. (2011) 311:15–8. 10.1016/j.jns.2011.08.046 21939984

[5] Holick MF. Vitamin D deficiency. N Engl J Med. (2007) 357:266–81. 10.1056/NEJMra070553 17634462

[6] Garbossa SG Folli F. Vitamin D, sub-inflammation and insulin resistance. A window on a potential role for the interaction between bone and glucose metabolism. Rev Endocr Metab Disord. (2017) 18:243–58. 10.1007/s11154-017-9423-2 28409320

[7] Mirhosseini N Vatanparast H Mazidi M Kimball SM. Vitamin D supplementation, glycemic control, and insulin resistance in prediabetics: a meta-analysis. J Endocr Soc. (2018) 2:687–709. 10.1210/js.2017-00472 29951596 PMC6016617

[8] Nagashima T Shirakawa H Nakagawa T Kaneko S. Prevention of antipsychotic-induced hyperglycaemia by vitamin D: a data mining prediction followed by experimental exploration of the molecular mechanism. Sci Rep. (2016) 6:26375. 10.1038/srep26375 27199286 PMC4873813

[9] Wenclewska S Szymczak-Pajor I Drzewoski J Bunk M Sliwińska A. Vitamin D supplementation reduces both oxidative DNA damage and insulin resistance in the elderly with metabolic disorders. Int J Mol Sci. (2019) 20:2891. 10.3390/ijms20122891 31200560 PMC6628266

[10] Gandhe MB Jain K Gandhe SM. Evaluation of 25(OH) vitamin D3 with reference to magnesium status and insulin resistance in T2DM. J Clin Diagn Res. (2013) 7:2438–41. 10.7860/JCDR/2013/6578.3568 24392366 PMC3879824

[11] Xu Z Gong R Luo G Wang M Li D Chen Y. Association between vitamin D3 levels and insulin resistance: a large sample cross-sectional study. Sci Rep. (2022) 12:119. 10.1038/s41598-021-04109-7 34997087 PMC8741779

[12] Erdönmez D Hatun S Çizmecioglu FM Keser A. No relationship between vitamin D status and insulin resistance in a group of high school students. J Clin Res Pediatr Endocrinol. (2011) 3:198–201. 10.4274/jcrpe.507 22155462 PMC3245493

[13] Vashum KP McEvoy M Milton AH Islam MR Hancock S Attia J. Is serum zinc associated with pancreatic beta cell function and insulin sensitivity in pre-diabetic and normal individuals? Findings from the Hunter Community Study. PLoS ONE. (2014) 9:e83944. 10.1371/journal.pone.0083944 24416185 PMC3885544

[14] Maret W. Zinc in pancreatic islet biology, insulin sensitivity, and diabetes. Prev Nutr Food Sci. (2017) 22:1–8. 10.3746/pnf.2017.22.1.1 28401081 PMC5383135

[15] Claro da Silva T Hiller C Gai Z Kullak-Ublick GA. Vitamin D3 transactivates the zinc and manganese transporter SLC30A10 via the Vitamin D receptor. J Steroid Biochem Mol Biol. (2016) 163:77–87. 10.1016/j.jsbmb.2016.04.006 27107558

[16] Fain JA. NHANES. Diabetes Educ. (2017) 43:151. 10.1177/0145721717698651 28340543

[17] Zipf G Chiappa M Porter KS Ostchega Y Lewis BG Dostal J. National health and nutrition examination survey: plan and operations, 1999–2010. Vital and health statistics. Ser 1 Prog Collect Proc. (2013) 1–37. 25078429

[18] Foster E Lee C Imamura F Hollidge SE Westgate KL Venables MC. Validity and reliability of an online self-report 24-h dietary recall method (Intake24): a doubly labelled water study and repeated-measures analysis. J Nutr Sci. (2019) 8:e29. 10.1017/jns.2019.20 31501691 PMC6722486

[19] Ahluwalia N Dwyer J Terry A Moshfegh A Johnson C. Update on NHANES dietary data: focus on collection, release, analytical considerations, and uses to inform public policy. Adv Nutr. (2016) 7:121–34. 10.3945/an.115.009258 26773020 PMC4717880

[20] Story MJ. Essential sufficiency of zinc, ω-3 polyunsaturated fatty acids, vitamin D and magnesium for prevention and treatment of COVID-19, diabetes, cardiovascular diseases, lung diseases and cancer. Biochimie. (2021) 187:94–109. 10.1016/j.biochi.2021.05.013 34082041 PMC8166046

[21] Lips P Eekhoff M van Schoor N Oosterwerff M de Jongh R Krul-Poel Y. Vitamin D and type 2 diabetes. J Steroid Biochem Mol Biol. (2017) 173:280–5. 10.1016/j.jsbmb.2016.11.021 27932304

[22] Heaney RP French CB Nguyen S Ferreira M Baggerly LL Brunel L. A novel approach localizes the association of vitamin D status with insulin resistance to one region of the 25-hydroxyvitamin D continuum. Adv Nutr. (2013) 4:303–10. 10.3945/an.113.003731 23674796 PMC3650499

[23] Burnett BP Pillai L Bitto A Squadrito F Levy RM. Evaluation of CYP450 inhibitory effects and steady-state pharmacokinetics of genistein in combination with cholecalciferol and citrated zinc bisglycinate in postmenopausal women. Int J Womens Health. (2011) 3:139–50. 10.2147/IJWH.S19309 21792336 PMC3140810

[24] Chailurkit LO Aekplakorn W Ongphiphadhanakul B. The association between vitamin D status and type 2 diabetes in a Thai population, a cross-sectional study. Clin Endocrinol. (2012) 77:658–64. 10.1111/j.1365-2265.2012.04422.x 22530700

[25] Al-Sofiani ME Jammah A Racz M Khawaja RA Hasanato R El-Fawal HA. Effect of vitamin D supplementation on glucose control and inflammatory response in type II diabetes: a double blind, randomized clinical trial. Int J Endocrinol Metab. (2015) 13:e22604. 10.5812/ijem.22604 25745497 PMC4338666

[26] Choi S Liu X Pan Z. Zinc deficiency and cellular oxidative stress: prognostic implications in cardiovascular diseases. Acta Pharmacol Sin. (2018) 39:1120–32. 10.1038/aps.2018.25 29926844 PMC6289396

[27] Du K Liu MY Zhong X Wei MJ. Decreased circulating Zinc levels in Parkinson's disease: a meta-analysis study. Sci Rep. (2017) 7:3902. 10.1038/s41598-017-04252-0 28634347 PMC5478669

[28] Frassinetti S Bronzetti G Caltavuturo L Cini M Croce CD. The role of zinc in life: a review. J Environ Pathol Toxicol Oncol. (2006) 25:597–610. 10.1615/JEnvironPatholToxicolOncol.v25.i3.40 17073562

[29] Gammoh NZ Rink L. Zinc in infection and inflammation. Nutrients. (2017) 9:624. 10.20944/preprints201705.0176.v1 PMC5490603 28629136

[30] Mendes Garrido Abregú F Gobetto MN Juriol LV Caniffi C Elesgaray R Tomat AL. Developmental programming of vascular dysfunction by prenatal and postnatal zinc deficiency in male and female rats. J Nutr Biochem. (2018) 56:89–98. 10.1016/j.jnutbio.2018.01.013 29525532

[31] Sahin O Elcik D Dogan A Cetinkaya Z Oguzhan A. The relation of serum trace elements and coronary atherosclerotic progression. Trace Elem Electrol. (2019) 36:210–4. 10.5414/TEX01570

[32] Samadi A Isikhan SY Tinkov AA Lay I Doşa MD Skalny AV. Zinc, copper, and oxysterol levels in patients with type 1 and type 2 diabetes mellitus. Clin Nutr. (2020) 39:1849–56. 10.1016/j.clnu.2019.07.026 31427180

[33] Morais JBS Severo JS Beserra JB de Oiveira ARS Cruz KJC de Sousa Melo RS. Association between cortisol, insulin resistance and zinc in obesity: a mini-review. Biol Trace Element Res. (2019) 191:323–30. 10.1007/s12011-018-1629-y 30617901

[34] Jansen J Karges W Rink L. Zinc and diabetes–clinical links and molecular mechanisms. The Journal of nutritional biochemistry (2009) 20:399–417. 10.1016/j.jnutbio.2009.01.009 19442898

[35] Barman S Srinivasan K. Zinc supplementation alleviates hyperglycemia and associated metabolic abnormalities in streptozotocin-induced diabetic rats. Can J Physiol Pharmacol. (2016) 94:1356–65. 10.1139/cjpp-2016-0084 27782759

[36] Nazarizadeh A Asri-Rezaie S. Comparative study of antidiabetic activity and oxidative stress induced by zinc oxide nanoparticles and zinc sulfate in diabetic rats. AAPS PharmSciTech. (2016) 17:834–43. 10.1208/s12249-015-0405-y 26349687

[37] Zofková I Nemcikova P Matucha P. Trace elements and bone health. Clin Chem Labor Med. (2013) 51:1555–61. 10.1515/cclm-2012-0868 23509220

[38] Yamaguchi M. Role of nutritional zinc in the prevention of osteoporosis. Mol Cell Biochem. (2010) 338:241–54. 10.1007/s11010-009-0358-0 20035439

[39] Meyer R De Koker C Dziubak R Godwin H Dominguez-Ortega G Shah N. Dietary elimination of children with food protein induced gastrointestinal allergy - micronutrient adequacy with and without a hypoallergenic formula? Clin Transl Allergy. (2014) 4:31. 10.1186/2045-7022-4-31 25328667 PMC4201676

[40] Oxentenko AS Murray JA. Celiac disease: ten things that every gastroenterologist should know. Clin Gastroenterol Hepatol. (2015) 13:1396–404. 10.1016/j.cgh.2014.07.024 25051511

[41] Santucci NR Alkhouri RH Baker RD Baker SS. Vitamin and zinc status pretreatment and posttreatment in patients with inflammatory bowel disease. J Pediatr Gastroenterol Nutr. (2014) 59:455–7. 10.1097/MPG.0000000000000477 25000354

[42] Allan K Devereux G. Diet and asthma: nutrition implications from prevention to treatment. J Am Diet Assoc. (2011) 111:258–68. 10.1016/j.jada.2010.10.048 21272700

[43] Uysalol M Uysalol EP Yilmaz Y Parlakgul G Ozden TA Ertem HV. Serum level of vitamin D and trace elements in children with recurrent wheezing: a cross-sectional study. BMC Pediatr. (2014) 14:270. 10.1186/1471-2431-14-270 25318349 PMC4286924

